# The removal mechanism and performance of tetrabromobisphenol A with a novel multi-group activated carbon from recycling long-root *Eichhornia crassipes* plants†

**DOI:** 10.1039/c9ra03374b

**Published:** 2019-08-09

**Authors:** Lili Liu, Xin Chen, Zhiping Wang, Xixi Wang, Sen Lin

**Affiliations:** State Environmental Protection Key Laboratory of Environmental Risk Assessment and Control on Chemical Process, East China University of Science and Technology Shanghai 200237 China linsen@ecust.edu.cn; National Engineering Research Center for Integrated Utilization of Salt Lake Resources, East China University of Science and Technology Shanghai China; School of Environment Science and Technology, Shanghai Jiao Tong University Shanghai China; Shanghai Institute of Pollution Control and Ecological Security Shanghai China

## Abstract

Long-root *Eichhornia crassipes* has shown great potential in eutrophication treatments while the heavy disposal of its plants limits its large-scale application. In this study, the adsorption of TBBPA by a novel multi-group activated carbon (MGAC), prepared from the reaped long-root *Eichhornia crassipes* plants has been investigated as a potential recycling and remediation technology. The MGAC showed great adsorption performance for aqueous TBBPA in that the adsorption could arrive at equilibrium in 4 h and the saturated adsorption capacities could reach up to 110.7, 110.5 and 75.50 mg g^−1^ at 20, 30 and 40 °C, respectively. Based on the analysis of adsorption processes, it was confirmed that π–π interaction and hydrogen bonding were the major impetuses for the adsorption and the oxygen-containing functional groups on the MGAC surface could facilitate the adsorption by either electron sharing or electron transfer. In addition, the thermodynamic results showed that the adsorption was a spontaneous and exothermic reaction. Futhermore, the MGAC could be regenerated easily by 5% NaOH solution and retained over 50% of its initial capacities for TBBPA after 5 reprocessing cycles. These results indicate the promising application of MGAC in the wastewater treatment for TBBPA removal and a resource recycling method for the long-root *Eichhornia crassipes* plants.

## Abstract 2

MGAC showed excellent adsorption performance for TBBPA and provide an ecologically friendly way to deal with eutrophic plants.

## Graphical abstract

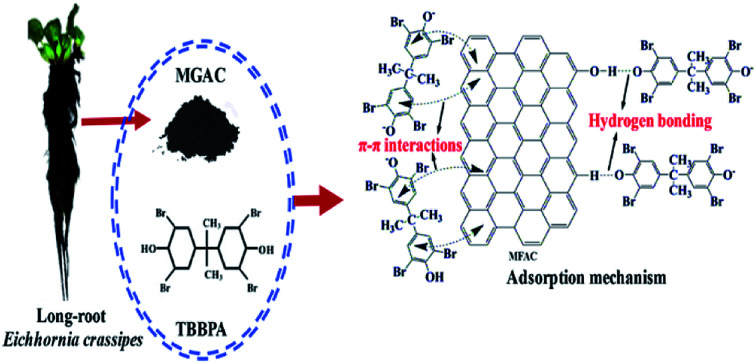

## 1. Introduction

Tetrabromobisphenol A (TBBPA) has been used extensively as a reactive or additive flame retardant in paper, textiles and plastics products, and especially for electronic products to reduce the flammability over the past decades.^1^ Up to now, TBBPA has been detected in various environmental samples, *i.e.* water,^2–4^ sediment,^5,6^ leachate,^7^*etc*. Besides TBBPA can pass through food chains and drinking water into the body, causing huge harm to human health owing to its endocrine disruption effects and possible carcinogenicity.^8^ Thus there have been growing efforts worldwide to remove TBBPA, including oxidation,^9,10^ reduction,^11,12^ pyrolysis^13^ and adsorption.^14–20^ Among them, adsorption by activated carbon has been one of the most extensively investigated methods due to its cheapness, effectiveness and convenience for subsequent operation.

At the same time, the high cost of raw materials (coconut shell, wood, coal, *etc.*) for commercial activated carbon leads to the high price of large-scale wastewater treatment using activated carbon. Therefore, the preparation of activated carbon from plant materials in natural environment has the advantages of low cost, environmental friendliness and rich structures. Among them, long-root *Eichhornia crassipes* with the characteristics of rapid growth and reproduction, wide distribution and easy access, has been planted to solve the eutrophication in Dianchi Lake in China dependent on its ultralong root, while how to dispose these ripe plants is still a great challenge. Therefore, it's essential and of great environmental significance to explore available approaches for the recycle of waste long-root *Eichhornia crassipes* plants for the practical applicability of this eutrophication treatment technology. According to the characteristics of the long-root *Eichhornia crassipes*, it would be comparatively profitable to transform and recycle them into the high-performance activated carbon for environmental contaminants. At present, most of the studies were focused on the adsorption of heavy metals by long-root *Eichhornia crassipes* activated carbon, while there are few reports on the adsorption of organic pollutants, which limited its further promotion.^21–23^ Therefore, it is necessary to increase the research on the adsorption mechanism of organic matters. In addition, it is extremely necessary to understand the underlying adsorption processes and mechanisms for the adsorption to further improving the practical performance of adsorbents.

In this study, a novel multi-group activated carbon (MGAC) was prepared from the long-root *Eichhornia crassipes* to investigate the adsorption performance and specific mechanism for aqueous TBBPA in the first time. After the preparation, the physicochemical characteristics of the MGAC were determined. Then the effects of different exogenous factors on adsorption were explored to deduce the correlative impetuses, including initial pH, ionic strength, *etc*. In addition, the adsorption dynamics and thermodynamics were described by multifarious models to analyze adsorption performance and mechanism. Finally, the regeneration and recycling of the MGAC were also investigated. Based on these results, the in-depth understanding of the adsorption mechanism and demonstration of the adsorption, regeneration and reuse of the MGAC was obtained and improved. It further indicated the feasibility of the practical application and recycling utilization of long-root *Eichhornia crassipes* plants.

## 2. Materials and methods

### 2.1 Chemicals

TBBPA used for adsorption was purchased from J&K Scientific Co., Ltd (Shanghai, China) with the purity over 98.0%. The other chemicals used in this work were all of analytically reagent grade and no further purification was performed prior to use. The TBBPA stock solution was prepared by dissolving 100 mg TBBPA into 100 ml 0.1% (m/m) NaOH solution. This solution was further diluted with deionized water to the concentration required for the following experiments. The long-root *Eichhornia crassipes* plants used to prepare the MGAC were reaped from the Dianchi Lake, Yunnan, China. In all experiments, the initial pH values of the solutions were adjusted by using 1% HCl or 1% NaOH solution.

### 2.2 Preparation of the MGAC

10.0 g powder of long-root *Eichhornia crassipes*, which had been dried and grinded, mixed with 30 ml 25% KOH solution under stirring for primary carbonization. Then 100 ml deionized water was added to impregnate the powder for 12 h. Afterwards, the powder was calcined at 600 °C for 1 h with nitrogen protection after dried at 100 °C. Finally, the MGAC could be collected by wash with 5% HCl to activate the calcined product.

### 2.3 Adsorption experiments

For a typical batch experiment, 15 mg MGAC was added to 100 ml TBBPA work solution with selected concentration at a given pH in a 250 ml flask. The flask was then placed in a temperature-controlled incubator shaker (YRH-300, Yaoshi Instrument, China) at 175 rpm at a given temperature. The supernatant was sampled at specified time intervals and filtered through a 0.22 μm filter for analysis. The adsorption capacity was calculated according to eqn (1).

1 *q*_e_ = (*C*_0_ − *C*_e_) × *V*/*m*

where *C*_0_ (mg L^−1^) and *C*_e_ (mg L^−1^) were the initial and equilibrium concentrations of the target contaminant, respectively. And *q*_e_ (mg g^−1^) was the adsorption capacity of the MGAC at equilibrium. *V* (*L*) was the contaminant solution volume and *m* (g) was the mass of the MGAC.

All experiments were performed in triplicate and the variations between parallel experiments were less than 5%. All glassware used were cleansed *via* sonication at 40 kHz for 30 min and then dried out before use.

### 2.4 Adsorption kinetics

The adsorption kinetic models were used to describe the adsorption process and the effect of contact time. 15 mg MGAC mixed with 100 ml TBBPA solutions of concentrations 5, 10 and 20 mg L^−1^ in 250 ml flask at pH 9.0, 175 rpm and 30 °C. The supernatant was sampled at specified time intervals and filtered through a 0.22 μm filter for analysis. All experiments were performed in triplicate and the variations between parallel experiments were less than 5%.

The pseudo-first-order and pseudo-second-order kinetic models can be expressed as (2) and (3), respectively:

2 *q*_*t*_ = *q*_e_(1 − e^−*k*_1_*t*^)

3 *q*_*t*_ = *k*_2_*q*_e_^2^*t*/(1 + *k*_2_*q*_e_*t*)

where *q*_*t*_ (mg g^−1^) is the adsorbed amount at time *t* (h), *q*_e_ (mg g^−1^) is the adsorbed amount at equilibrium, *k*_1_ (h^−1^), *k*_2_ (g (mg h)^−1^) are the pseudo-first-order and pseudo-second-order rate constant, respectively.

The intra-particle diffusion equation is:

4 *q*_*t*_ = *k*_ip_*t*^1/2^ + *C*

where *k*_ip_ [mg (g h^1/2^)^−1^] is the rate constant in the intra-particle diffusion equation. The boundary layer thickness is determined by *C*. If the plot of *q*_*t*_ (mg g^−1^) *vs. t*^1/2^ (h^1/2^) is a straight line and passes through the origin, the sole factor controlling the adsorption ratio is intra-particle diffusion.^24^

### 2.5 Adsorption isotherms

The adsorption isotherm models were used to determine the adsorption mechanism and describe how TBBPA molecules interact with the MGAC. 100 ml TBBPA solutions of five concentrations, *i.e.*, 2, 5, 10, 20, 30 mg L^−1^, were prepared and used in adsorption isotherm experiments. The supernatant was sampled at specified time intervals and filtered through a 0.22 μm filter for analysis. All experiments were performed in triplicate and the variations between parallel experiments were less than 5%.

Langmuir isotherm assumes that the adsorbate is uniformly adsorbed on the surface of the adsorbent. The adsorption process is monolayer and the adsorbate does not move on the surface.

5 *C*_e_/*Q*_e_ = 1/(*Q*_m_*K*_L_) + *C*_e_/*Q*_m_

where *Q*_e_ (mg g^−1^) is the equilibrium adsorbance, *C*_e_ (mg L^−1^) is the equilibrium concentration of TBBPA in the solution, *Q*_m_ (mg g^−1^) is the saturation adsorption capacity, *K*_L_ (L mg^−1^) is the Langmuir model constant.

The Freundlich models indicates that multilayer adsorption is carried out on a heterogeneous surface, which was expressed by eqn (6).6
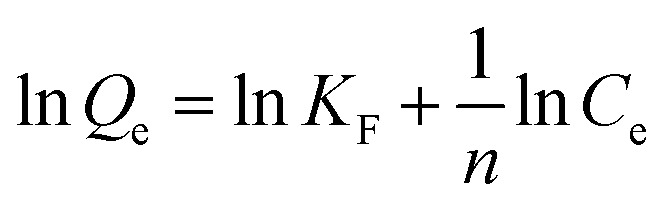
where *Q*_e_ (mg g^−1^) is the equilibrium adsorbance, *C*_e_ (mg L^−1^) is the equilibrium concentration of TBBPA in the solution, *Q*_m_ (mg g^−1^) is the saturation adsorption capacity, *K*_F_ and *n* are the Freundlich model constants.

The Temkin model is mainly used to describe the chemical adsorption process. It is considered that the adsorption heat varies linearly with temperature. The equation is as follows:

7 *Q*_e_ = *B*_1_ ln *K*_T_ + *B*_1_ ln *C*_e_

where *Q*_e_ (mg g^−1^) is the equilibrium adsorbance, *C*_e_ (mg L^−1^) is the equilibrium concentration of TBBPA in the solution, *B*_1_ is the reaction heat of adsorption, *K*_T_ (L mg^−1^) is the Temkin model constants.

The Dubinin–Radushkevich (D–R) model equation is as follows:

8 ln *Q*_e_ = ln *Q*_m_ − *kε*^2^

9 *ε* = *RT* ln(1 + 1/*C*_e_)

where *Q*_e_ (mg g^−1^) is the equilibrium adsorbance, *C*_e_ (mg L^−1^) is the equilibrium concentration of TBBPA in the solution, *k* (mol^2^ J^−2^) is a constant that corresponds to the adsorption energy, *ε* is the Polanyi potential, *R* is the ideal gas constant [8.314 J (mol K)^−1^], *Q*_m_ (mg g^−1^) is the maximum adsorption capacity.

In addition, the adsorption thermodynamics parameters including standard enthalpy (Δ*H*^0^, kJ mol^−1^), standard entropy (Δ*S*^0^, J (mol K)^−1^) and Gibbs free energy (Δ*G*^0^, kJ mol^−1^) were calculated according to eqn (10) and (11).10
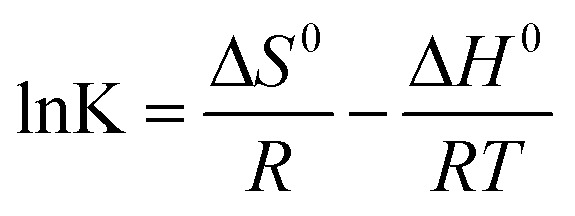

11 Δ*G*^0^ = Δ*H*^0^ − *T*Δ*S*^0^

where *K* (L g^−1^) is the adsorption equilibrium constant, *R* (8.314 J (mol K)^−1^) is the universal gas constant, *T* is the temperature (K).

### 2.6 Regeneration

1.0 g of the used MGAC mixed with 30 ml 5% NaOH solution in a 150 ml flask and then the flask was placed in incubator shaker at 30 °C and 175 rpm for 2 h. At last the spent MGAC was washed with deionized water until the solution pH reached 6.0–7.0. After the regeneration, the remaining TBBPA in the solution was measured, and the regenerated MGAC was applied for the subsequent adsorption cycles.

### 2.7 Characterization methods

The TEM and SEM images of the MGAC were examined on an transmission electron microscope (JEM-2100, JEOL, Japan) with a high voltage of 200 kV and an scanning electron microscopy (JSM-6360LV, JEOL, Japan) with 5.0 kV, respectively. The specific surface area was calculated by the Brunauer–Emmett–Teller (BET) method and the pore volume and pore size distribution were estimated based on Barrett–Joyner–Halenda (BJH) model. The X-ray photoelectron spectroscopy (XPS) spectrum of this adsorbent was obtained by an XPS instrument (ESCALAB 250Xi, Thermo Scientific, USA) with the Al-KR as the excitation source. The zeta potentials of the MGAC were determined by a zeta potential instrument (Nano ZS, Malvern, UK) following the method of Wang *et al.*^25^ Fourier-transform infrared (FTIR) spectra of the MGAC before and after adsorption were recorded from samples in the wavenumber range of 4000–400 cm^−1^ in KBr pellets on a FTIR spectrometer (NICOLET 6700, Thermofisher, USA). The concentrations of TBBPA in solutions were determined by a high performance liquid chromatography (HPLC) (LC-20AT, Shimadzu, Japan) equipped with an UV detector and a C18 reverse-phase column (Inertsil ODS) (250 mm × 4.6 mm i.d., particle size 5 μm) using methanol/water (80 : 20 (v/v)) mobile phase. The pH values of solutions were measured with a pH meter (HQ30d, HACH, USA) after calibration.

## 3. Results and discussion

### 3.1 Characterization of the MGAC

**Fig. 1:**
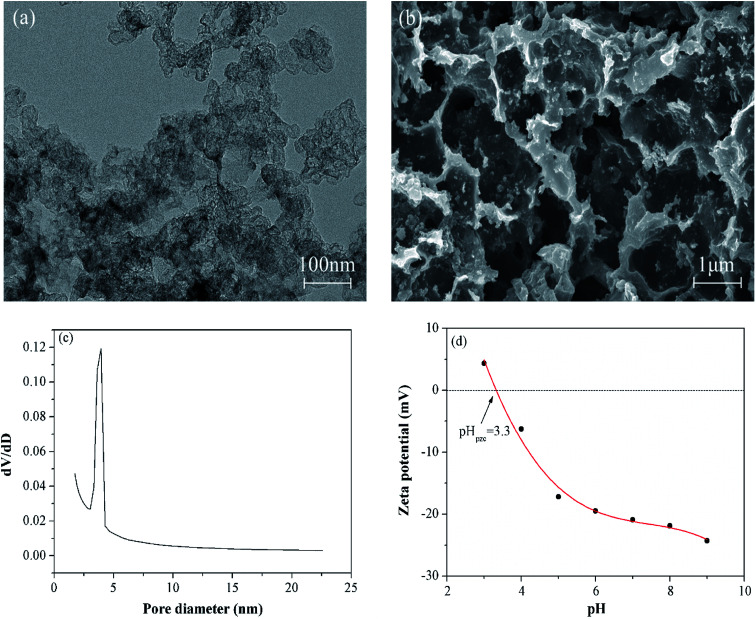
The SEM (a) and TEM (b) images of the MGAC; the pore size distribution (c) of the MGAC; the zeta potential of the MGAC as a function of pH (d).

In this study, multiple measurements were applied to characterize the physicochemical properties of the prepared MGAC. Firstly, the morphology and microstructure of the MGAC were demonstrated by the TEM and SEM images (Fig. 1). The TEM observation of the MGAC indicated that the carbon had significantly hierarchical porous structures with honeycomb-like shape (Fig. 1a). And the SEM (Fig. 1b) observation presented a rough and irregular surface with pores on the MGAC. According to the BET result, it might be related to the porosity of the adsorbent, thus resulted in a high specific surface area^26,27^ reaching than 874.3 m^2^ g^−1^. The pore size distribution curve (Fig. 1c) exhibited in the category of mesopore, with the pore volume and average size being at 0.38 cm^3^ g^−1^ and 6.96 nm, respectively. In addition, the isoelectric point of the MGAC was identified from variation trend of the zeta potential as a function of pH values. As shown in Fig. 1d, the zeta potential decreased with the increasing pH, from which the isoelectric point was determined as 3.3, indicating the MGAC surface was electronegative in non-acid condition.

**Fig. 2:**
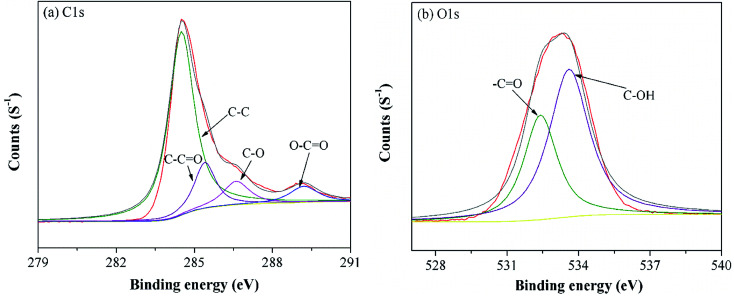
High resolution XPS spectra of C1s (a) and O1s (b) on the MGAC surface.

To further understand the functional groups on the MGAC surface, the wide XPS spectrum of the MGAC was surveyed (Fig. S1†), implying the oxygen, carbon and nitrogen were the dominant elements. To further explore the surface carbon and oxygen functional components, the high-resolution spectrum of C1s and O1s were deconvoluted by XPSPEAK41 software and the results were plotted in Fig. 2. And the C1s peak could be decomposed into four individual peaks at 284.6, 285.4, 286.6 and 289.2 eV (Fig. 2a), which were responsible for C–C in aromatic rings, C–C=O, C–O and O–C=O, respectively.^28,29^ In the case of oxygen in Fig. 2b, there were also two distinct oxygen bands at 532.4, and 533.6 eV, which were ascribed to C–O and C–OH, respectively, consistent with previous work.^14^ The proportions of functional groups both in C1s and O1s peak indicated that there were abundant oxygen-containing functional groups on the MGAC surface, affording great potential for the adsorption (Table S1†).

### 3.2 Effects of environmental factors on the adsorption of TBBPA

#### 3.2.1 Initial pH

**Fig. 3:**
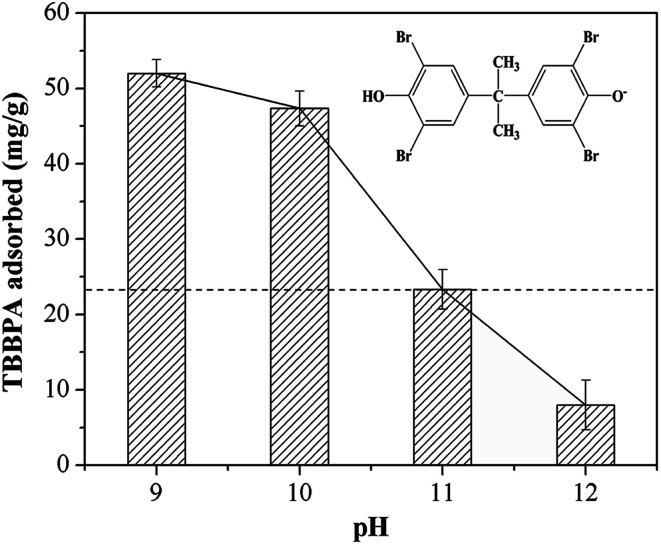
Effect of initial pH on TBBPA adsorption using the MGAC (TBBPA = 10 mg L^−1^, MGAC = 150 mg L^−1^, *T* = 30 °C, *t* = 10 h).

pH is one of the vital factors affecting the adsorption performance, which determines the surface charge on the adsorbent surface as well as the existing speciation of adsorbate in solution. The experiments were conducted with initial pH value varying in the range of 9.0–12.0, in view of the insolubility of TBBPA in non-alkaline solution.^30^ As shown in Fig. 3, TBBPA adsorbed on the MGAC decreased from 52.1 mg g^−1^ to 8.0 mg g^−1^ with the increase of initial pH in solution from 9.0 to 12.0, indicating the alkaline condition unfavorable for the adsorption. According to the distribution of molecular and anionic forms as a function of pH, illustrated in Fig. S2,† TBBPA was mostly ionized to mono or divalent anions in the alkaline condition.^18^ As the pH value exceeded 9.0, these functional groups on the MGAC surface were deprotonated, such as –COO^−^, –O^−^, *etc*., resulting in the decrease of the adsorption capacity due to the enhanced electrostatic repulsion between the MGAC surface and TBBPA. In addition, attributed to the reduction of hydrogen bonding in strongly basic solution, which was prevented by the enhanced charge repulsion, TBBPA adsorption decreased severely when pH increased from 10.0 to 12.0.

#### 3.2.2 Ionic strength

**Fig. 4:**
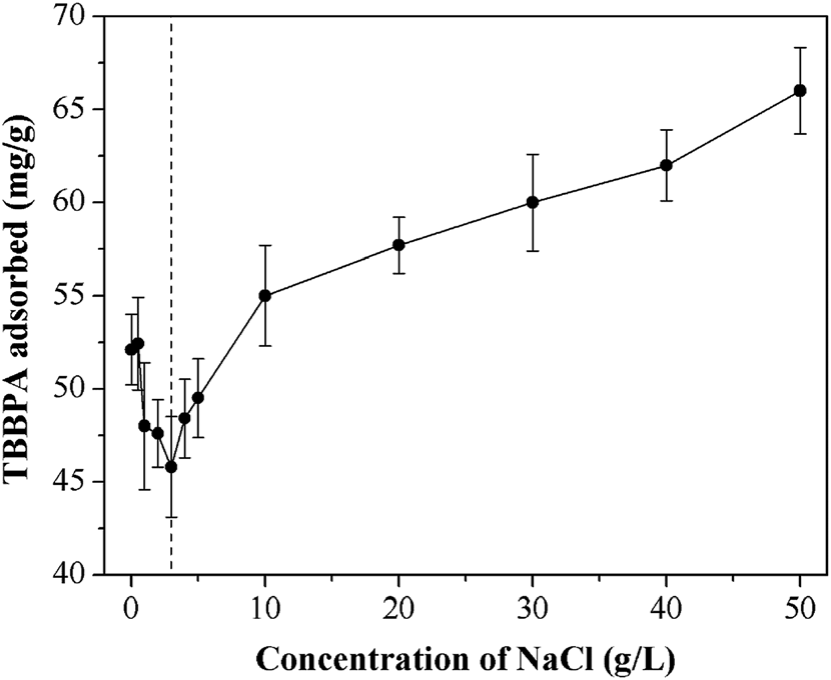
Effect of ionic strength on TBBPA adsorption using the MGAC (TBBPA = 10 mg L^−1^, MGAC = 150 mg L^−1^, *T* = 30 °C, pH = 9.0, *t* = 10 h).

In this study, the effects of ionic strength on TBBPA adsorption using the MGAC was performed with the concentrations of NaCl at the range of 0–50 g L^−1^. The adsorption capacity decreased slightly with NaCl concentration in the range of 0–3 g L^−1^ (Fig. 4), due to that the electrostatic repulsion would be reinforced under a small amount of NaCl in solution. On the contrary, the adsorption capacity of TBBPA increased from 48.4 mg g^−1^ to 66.0 mg g^−1^ with the increase of NaCl concentration in 3–50 mg L^−1^. According to the results of Amrit *et al.*,^31^ the increased adsorption capacity might come from the π–π interaction between TBBPA and the MGAC, which could be effectively enhanced by stronger ionic strength. In addition, Na^+^ bridged with negatively charged surface groups might promote the adsorption resulting from offsetting the negative effects of competing adsorption sites.^32^

#### 3.2.3 Humic acid

**Fig. 5:**
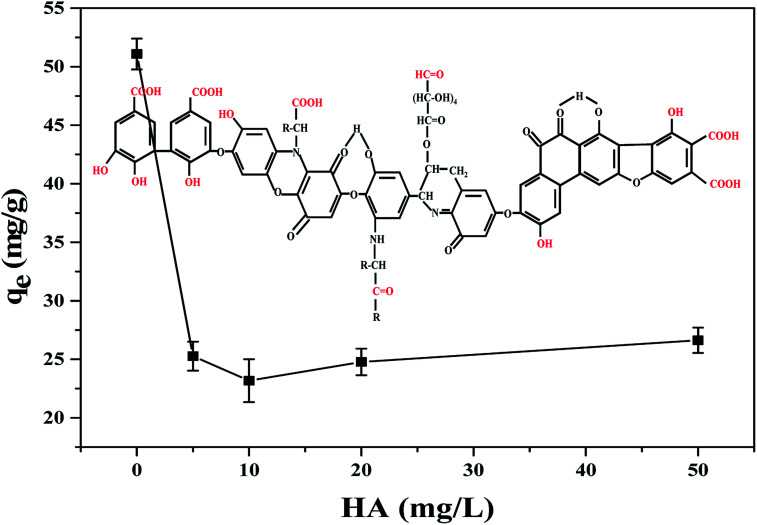
Effect of HA on TBBPA adsorption using the MGAC (TBBPA = 10 mg L^−1^, MGAC = 150 mg L^−1^, HA = 0, 5, 10, 20, 50 mg L^−1^, *T* = 30 °C, pH = 9.0, *t* = 10 h).

Humic acid (HA) is ubiquitous in natural waters derived by the microbial degradation of dead plants, which is always concomitant with TBPPA.^33^ Hence, it's full of importance to investigate the effect of the coexistence of HA on TBBPA adsorption. According to results of TBBPA adsorption at different concentrations of HA (Fig. 5), the adsorption capacity decreased significantly with HA concentration less than 10 mg L^−1^ while the adsorbed TBBPA was enhanced as HA concentration continued to increase. The basic structure of HA is aromatic and alicyclic ring, connected with carboxyl, carbonyl, quinonyl, hydroxyl and methoxy groups.^34^ All these structure can form the π–π interaction, hydrogen bonding with MGAC, which could attributed to the competitive adsorption of HA and TBBPA on adsorbents thus reduce the removal efficiency of TBBPA.^35,36^ The slight increase in adsorption at high concentration of HA was presumably due to the adsorption of free TBBPA to HA which was sorbed to the MGAC surface.^37^

### 3.3 Adsorption kinetics

**Fig. 6:**
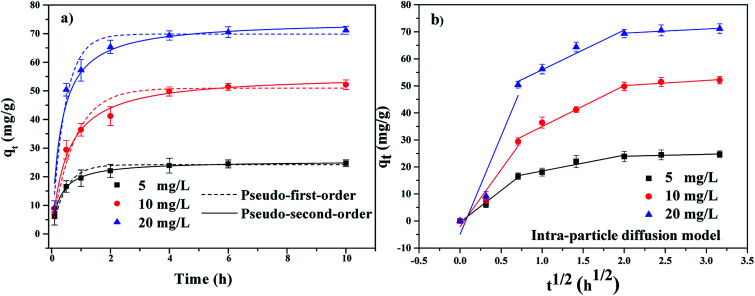
The adsorption kinetics of TBBPA on the MGAC (TBBPA = 5, 10 and 20 mg L^−1^, MGAC = 150 mg L^−1^, *T* = 30 °C, pH = 9.0, *t* = 10 h).

The adsorption kinetics of TBBPA using the MGAC was conducted under the initial concentrations of 5, 10 and 20 mg L^−1^. As shown in Fig. 6, it could be seen that the TBBPA adsorped on the MGAC surface all increased quickly in the first 2 h owing to the rich availability of active sites, and then the adsorption rate slowed down until the adsorption equilibrium reached in 4 h. In order to further analyze the adsorption processes, the pseudo-first-order and pseudo-second-order kinetic models were both used to fit the adsorption kinetic data shown in Fig. 6a. It could been seen that the adsorption was all better described by the pseudo-second-order model for different initial concentrations, which was verified by the fitting coefficients listed in Table 1 higher than 0.96. According to the hypothesis of the pseudo-second-order model, it indicated that the adsorption rate was controlled by chemical interaction, and there might be electron exchange or covalent bond between adsorbents and adsorbates. Besides the adsorption capacity was proportional to the functional groups involving π–π bonding and hydrogen bonding interactions on the absorbent, consistent with the studies of Zhang *et al.*^18^

Kinetic fitting parameters for TBBPA adsorption on the MGAC

**Table:** 

*C*_0_ (mg L^−1^)	Pseudo-first-order			Pseudo-second-order		
	*k*_1_ (h^−1^)	*q*_e_ (mg g^−1^)	*R* ^2^	*k*_2_ g (mg h)^−1^	*q*_e_ (mg g^−1^)	*R* ^2^
5	2.164	24.20	0.9567	0.137	25.48	0.9974
10	1.329	50.93	0.9800	0.033	55.70	0.9940
20	2.223	69.84	0.9559	0.041	74.57	0.9623

**Table:** 

Intra-particle diffusion model									
*C*_0_ (mg L^−1^)	First step			Second step			Third step		
	*K*_ip 1_ (mg g^−1^ h^−0.5^)	*C*_1_ (mg g^−1^)	*R* ^2^	*K*_ip 2_ (mg g^−1^ h^−0.5^)	*C*_2_ (mg g^−1^)	*R* ^2^	*K*_ip 3_ (mg g^−1^ h^−0.5^)	*C*_3_ (mg g^−1^)	*R* ^2^
5	2.5451	−0.4788	0.9839	5.8897	12.5903	0.9259	0.7023	22.5618	0.7274
10	42.1574	−2.0828	0.9085	15.1477	19.8061	0.9766	1.9223	46.2472	0.7555
20	72.6213	−4.9208	0.8346	14.6827	41.2785	0.9382	1.5370	66.4384	0.8304

The plots of *q*_*t*_*vs. t*^1/2^ at different initial concentrations are shown in Fig. 6b. It could been seen that the plots are not linear and do not pass through the origin, which demonstrated that the adsorption was a complicated process including multiple steps.^38^Fig. 6b clearly shown that the adsorption could be divided into three steps. The first step was the instantaneous adsorption or external surface adsorption in the range of 0–0.5 h and approximately 50% of the adsorption capacities finished in this step mainly relying on the high concentration of TBBPA as the adsorption impetus.^39^ The second step was controlled by the intra-particle diffusion and the curve represented gradual adsorption, where TBBPA was removed by the internal sites.^24^ In the final equilibrium stage, the adsorption growth rate was almost constant and TBBPA concentration kept at a low level, indicating the adsorption equilibrium arriving.^40^

### 3.4 Adsorption thermodynamics

**Fig. 7:**
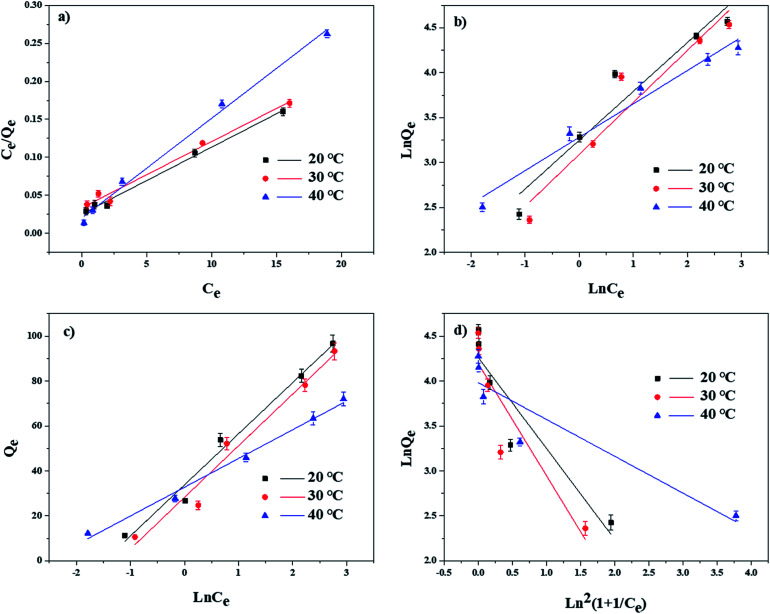
Adsorption isotherms of TBBPA on MGAC in different temperature as well as modeling using the (a) Langmuir, (b) Freundlich, (c) Temkin and (d) D–R equations (TBBPA = 2, 5, 10, 20 and 30 mg L^−1^, MGAC = 150 mg L^−1^, *T* = 20, 30 and 40 °C, pH = 9.0, *t* = 10 h).

To further understand the adsorption mechanisms of TBBPA by the MGAC, adsorption isotherms were determined at temperature being at 20, 30 and 40 °C with various models of Langmuir, Freundlich, Temkin and D–R equations, respectively (Fig. 7). The corresponding models and fitting parameters were given in Table 2. Among the different models, the Langmuir model, with the higher correlation coefficient values (*R*^2^ > 0.981), could describe the adsorption better than the other isotherm models, indicating the existence of monolayer adsorption in the TBBPA removal process. According to the fitting results, the saturated adsorption capacity of the MGAC for TBBPA came up to 110.7 mg g^−1^, which was significantly superior to common commercial activated carbons (Fig. S3†). Moreover, the *n* values of Freundlich model ranged from 1.726 to 2.691 indicated that this adsorption process was feasible and all adsorption isotherms were nonlinear,^25^ which could be attributed to the various functional groups on the MGAC and the different dominant interactions like hydrogen bonding in the adsorption process.

**Table:** Isotherm parameters for TBBPA adsorption on MGAC

*T* (°C)	Langmuir			Freundlich			Temkin			*D*–*R*		
	*Q*_m_ (mg g^−1^)	*K*_L_ (L mg^−1^)	*R* ^2^	*K*_F_	*n*	*R* ^2^	*B*_1_	*K*_T_	*R* ^2^	*Q*_m_	*k*	*R* ^2^
20	110.7	0.3919	0.993	25.768	1.839	0.907	22.750	4.422	0.978	71.0	1.012	0.830
30	110.2	0.3070	0.981	21.979	1.726	0.902	22.969	3.423	0.962	66.5	1.250	0.792
40	75.5	0.6156	0.993	26.618	2.691	0.969	12.794	12.931	0.990	53.7	0.410	0.807

In addition, the thermodynamics parameters including Δ*G*^0^, Δ*H*^0^ and Δ*S*^0^ were also calculated and listed in Table 3. The results showed that Δ*G*^0^ was negative at different temperatures, indicating that the TBBPA adsorption using the MGAC was a thermodynamically feasible and spontaneous process. The negative Δ*H*^0^ illustrated that the adsorption was an exothermic process, which would present preferable performance with lower temperature.

**Table:** Thermodynamic parameters for TBBPA adsorption on the MGAC at different temperatures

*T* (°C)	Δ*G*^0^ (kJ mol^−1^)	Δ*H*^0^ (kJ mol^−1^)	Δ*S*^0^ (J mol^−1^ K^−1^)
20	−18.79	−4.97	47.16
30	−19.26		
40	−19.76		

### 3.5 Adsorption mechanism of the MGAC for TBBPA

**Fig. 8:**
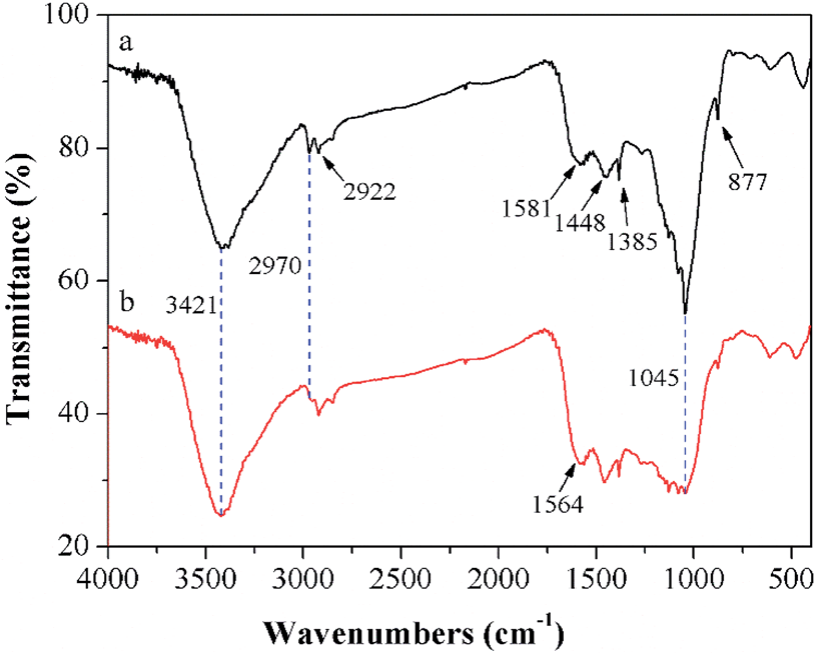
FTIR spectra of the MGAC (a) and the MGAC with TBBPA adsorbed (b).

According to previous studies, diversified adsorption impetuses to remove TBBPA using activated carbons had been proposed, including electrostatic interactions, hydrogen bonding, π–π interactions, hydrophobic effect.^32^ To further understand the adsorption mechanisms of TBBPA onto the MGAC surface, the FTIR spectra of the MGAC before and after adsorption were compared (Fig. 8). In addition, the peaks corresponding functional groups were demonstrated which certified the existence of multifarious oxygen-containing groups on the MGAC surface (Table 4). It is worth to highlight the change of the band at 1045 cm^−1^ after adsorption, which could be ascribed to the combination between TBBPA and –OH groups on the MGAC surface *via* hydrogen bonding.^17^ The disappearance of band at 2922 cm^−1^ and shift of band at 1581 cm^−1^ to 1564 cm^−1^ after adsorption could be attributed to the π–π interactions between TBBPA and the MGAC resulting in the variations of electron density and on the MGAC surface.^18^

**Table:** The peaks of FTIR spectra corresponding to functional groups

Peak (cm^−1^)	Characteristic vibration	Functional groups
3421	O–H vibration	–OH^41^
2970	O–H vibration	–OH^42^
2922	C–H stretching vibration	–CH_*x*_^17^
1581	Stretching vibrations of aromatic rings	Aromatic rings^43^
1448	Stretching vibrations of aromatic rings	Aromatic rings^43^
1385	O–H bending vibration	–OH^17^
1045	C–O stretching vibration	C=O^44^
877	C=C vibration	C=C^17^

**Fig. 9:**
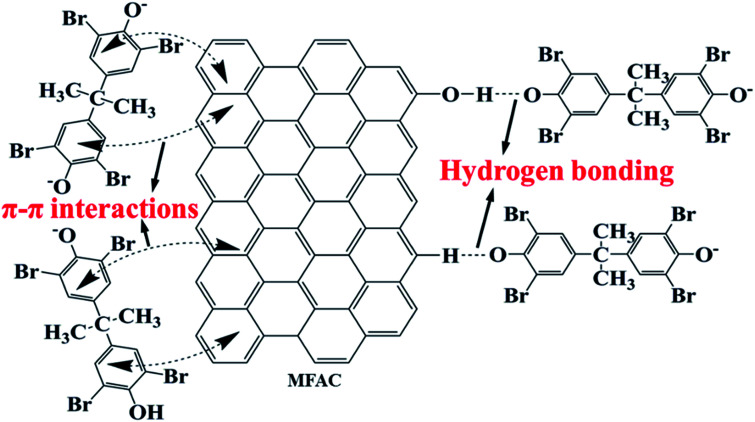
Schematic diagram for the adsorption of TBBPA using the MGAC.

Based on these results, the possible adsorption processes of TBBPA onto the MGAC surface were further proposed (Fig. 9). It was deduced that the π–π interactions probably played a significant role in the adsorption due to the benzene rings and –OH groups on the MGAC surface as the electron-donating functional groups might enhance the π-donating strength of the aromatic rings of TBBPA. In addition, hydrogen bonding also generated significant influence on the adsorption for the MGAC could act as hydrogen bond donors dependent on the –OH groups and aromatic rings on the surface, resulting in a better adsorption capacity. Besides, there may be electron exchange or covalent bond between adsorbents and adsorbates according to the results of kinetic model analysis.

### 3.6 Regeneration of MGAC

**Fig. 10:**
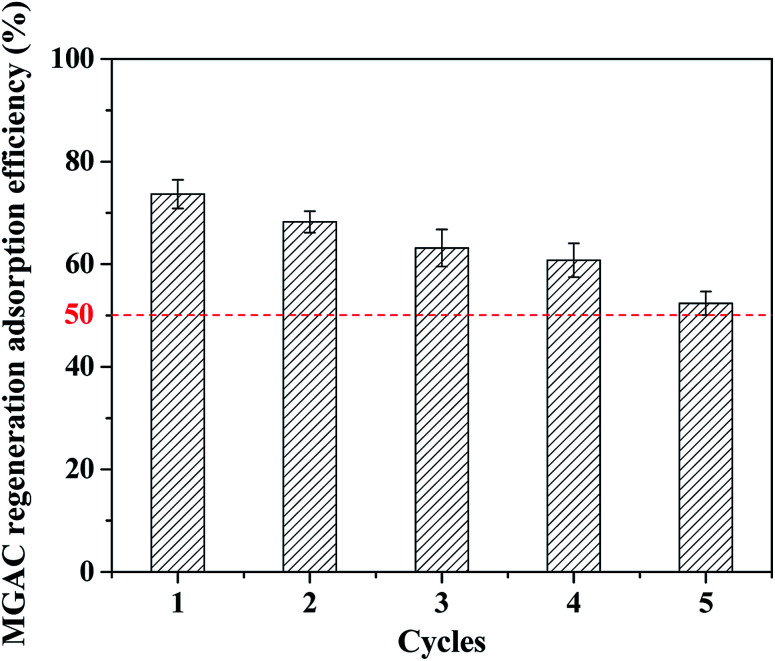
Regeneration of MGAC by 5% NaOH (TBBPA = 10 mg L^−1^, MGAC = 150 mg L^−1^, *T* = 30 °C, pH = 9.0, *t* = 10 h).

In order to extend the lifetime of the MGAC, the used MGAC was regenerated using NaOH solution as the eluent and then subjected to the TBBPA adsorption again. As shown in Fig. 10, the spent MGAC was successfully regenerated by alkaline and retained over 50% of its initial capacity for TBBPA after 5 successive adsorption–regeneration cycles, which could be attributed to a decrease in the number of adsorption sites. The results nevertheless proved that the MGAC based the long-root *Eichhornia crassipes* could be used repeatedly in an adsorption–desorption cycle, which was an essential advantage with regard to the practical applications. In addition, many regenerative methods have been developed recently, such as pyrolysis, which is versatile because it can decompose various organic contaminants. Studies have confirmed that TBBPA can be pyrolyzed at 280–900 °C,^45^ which provides a new efficient strategy for the MGAC regeneration in the future application.

## 4. Conclusion

In this study, the MGAC prepared from the waste long-root *Eichhornia crassipes* plants, had abundant functional groups on its surface, which could effectively remove TBBPA, indicating that it had good adsorption performance for organic pollutants. Moreover, it was confirmed that π–π interaction and hydrogen bonding were the major impetuses for the adsorption on the basis of multiple characterization and experimental analysis. The in-depth understanding of the adsorption properties and mechanism will offer valuable counsel for the application of the MGAC in the wastewater treatment and provide a new way for the resource utilization of long-root *Eichhornia crassipe* in the future.

## Conflicts of interest

There are no conflicts to declare.

## Supplementary Material

RA-009-C9RA03374B-s001

## References

[cit1] Lyche J. L., Rosseland C., Berge G., Polder A. (2015). Environ. Int..

[cit2] Xu J., Zhang Y., Guo C., He Y., Li L., Meng W. (2013). Environ. Toxicol. Chem..

[cit3] Xiong J., An T., Zhang C., Li G. (2015). Environ. Geochem. Health.

[cit4] Yang Y., Lu L., Zhang J., Yang Y., Wu Y., Shao B. (2014). J. Chromatogr. A.

[cit5] Feng A. H., Chen S. J., Chen M. Y., He M. J., Luo X. J., Mai B. X. (2012). Mar. Pollut. Bull..

[cit6] Lu Z., Letcher R. J., Chu S., Ciborowski J. J. H., Douglas Haffner G., Drouillard K. G., MacLeod S. L., Marvin C. H. (2015). J. Great Lakes Res..

[cit7] Li F. T., Yang T. (2017). Automatica.

[cit8] Grosse Y., Loomis D., Guyton K. Z., El Ghissassi F., Bouvard V., Benbrahim-Tallaa L., Mattock H., Straif K. (2016). Lancet Oncol..

[cit9] Ji Y., Kong D., Lu J., Jin H., Kang F., Yin X., Zhou Q. (2016). J. Hazard. Mater..

[cit10] Bao Y., Niu J. (2015). Chemosphere.

[cit11] Huang Q., Liu W., Peng P., Huang W. (2013). Chemosphere.

[cit12] Huang Q., Liu W., Peng P., Huang W. (2013). J. Hazard. Mater..

[cit13] Altarawneh M., Dlugogorski B. Z. (2014). J. Phys. Chem. A.

[cit14] Zhou L., Ji L., Ma P. C., Shao Y., Zhang H., Gao W., Li Y. (2014). J. Hazard. Mater..

[cit15] Tong F., Gu X., Gu C., Ji R., Tan Y., Xie J. (2015). Sci. Total Environ..

[cit16] Fasfous I. I., Radwan E. S., Dawoud J. N. (2010). Appl. Surf. Sci..

[cit17] Shen J., Huang G., An C., Xin X., Huang C., Rosendahl S. (2018). Bioresour. Technol..

[cit18] Zhang Y., Tang Y., Li S., Yu S. (2013). Chem. Eng. J..

[cit19] Sun Z., Yu Y., Mao L., Feng Z., Yu H. (2008). J. Hazard. Mater..

[cit20] Zhang Y., Jing L., He X., Li Y., Ma X. (2015). J. Ind. Eng. Chem..

[cit21] Cao F., Lian C., Yu J., Yang H., Lin S. (2019). Bioresour. Technol..

[cit22] Lin S., Yang H., Na Z., Lin K. (2017). Chemosphere.

[cit23] Lin S., Wang G., Na Z., Lu D., Liu Z. (2012). Chem. Eng. J..

[cit24] Srihari V., Das A. (2008). Desalination.

[cit25] Wang W., Deng S., Li D., Ren L., Shan D., Wang B., Huang J., Wang Y., Yu G. (2018). Chem. Eng. J..

[cit26] Song M., Jin B., Xiao R., Yang L., Wu Y., Zhong Z., Huang Y. (2013). Biomass Bioenergy.

[cit27] Ould-Idriss A., Stitou M., Cuerda-Correa E. M., Fernández-González C., Macías-García A., Alexandre-Franco M. F., Gómez-Serrano V. (2011). Fuel Process. Technol..

[cit28] Wang J., Liu T.-L., Huang Q.-X., Ma Z.-Y., Chi Y., Yan J.-H. (2017). Fuel Process. Technol..

[cit29] Gong Z., Li S., Ma J., Zhang X. (2016). Sep. Purif. Technol..

[cit30] Malkoske T., Tang Y., Xu W., Yu S., Wang H. (2016). Sci. Total Environ..

[cit31] Kalra A., Tugcu N., Cramer S. M., Garde S. (2001). J. Phys. Chem. B.

[cit32] Kah M., Sigmund G., Xiao F., Hofmann T. (2017). Water Res..

[cit33] Lv Y. Y., Yu X. H., Tu S. T., Yan J. Y., Dahlquist E. (2012). Appl. Energy.

[cit34] Jiang L. H., Liu Y. G., Zeng G. M., Xiao F. Y., Hu X. J., Hu X., Wang H., Li T. T., Zhou L., Tan X. F. (2016). Chem. Eng. J..

[cit35] Zhang Y., Tang Y., Li S., Yu S. (2013). Chem. Eng. J..

[cit36] Shuai L., Wu P., Chen M., Yu L., Kang C., Zhu N., Zhi D. (2017). Environ. Pollut..

[cit37] Behera S. K., Oh S. Y., Park H. S. (2010). J. Hazard. Mater..

[cit38] Qiang K., Xiao H., Li S., Sheng M. M. (2017). Process Saf. Environ. Prot..

[cit39] Liu L., Hu S., Shen G., Farooq U., Zhang W., Lin S., Lin K. (2018). Chemosphere.

[cit40] Depci T., Kul A. R., Önal Y. (2012). Chem. Eng. J..

[cit41] Al-Lagtah N. M. A., Al-Muhtaseb A. a. H., Ahmad M. N. M., Salameh Y. (2016). Microporous Mesoporous Mater..

[cit42] Islam M. A., Ahmed M. J., Khanday W. A., Asif M., Hameed B. H. (2017). Ecotoxicol. Environ. Saf..

[cit43] Deng H., Li G., Yang H., Tang J., Tang J. (2010). Chem. Eng. J..

[cit44] Chang K.-L., Hsieh J.-F., Ou B.-M., Chang M.-H., Hseih W.-Y., Lin J.-H., Huang P.-J., Wong K.-F., Chen S.-T. (2012). Sep. Sci. Technol..

[cit45] Altarawneh M., Saeed A., Al-Harahsheh M., Dlugogorski B. Z. (2019). Prog. Energy Combust. Sci..

