# Supplementary material for: The removal mechanism and performance of tetrabromobisphenol A with a novel multi-group activated carbon from recycling long-root Eichhornia crassipes plants
Source: RSC Adv. 2019 Aug 9;9(43):24760–9. doi: 10.1039/c9ra03374b (PMC9069885; doi:10.1039/c9ra03374b)
Supplement: RA-009-C9RA03374B-s001 [file RA-009-C9RA03374B-s001.pdf]

Electronic Supplementary Material (ESI) for RSC Advances.
This journal is © The Royal Society of Chemistry 2019

Supporting information

Table S1 The proportions of functional groups both in C1s and O1s peak.

Peak	Functional groups	Proportion/%
C1s	C-C in aromatic rings	51.4
	C-C=O	34.2
	C-O	6.8
	O-C=O	7.5
O1s	C-O	57.1
	C-OH	42.9

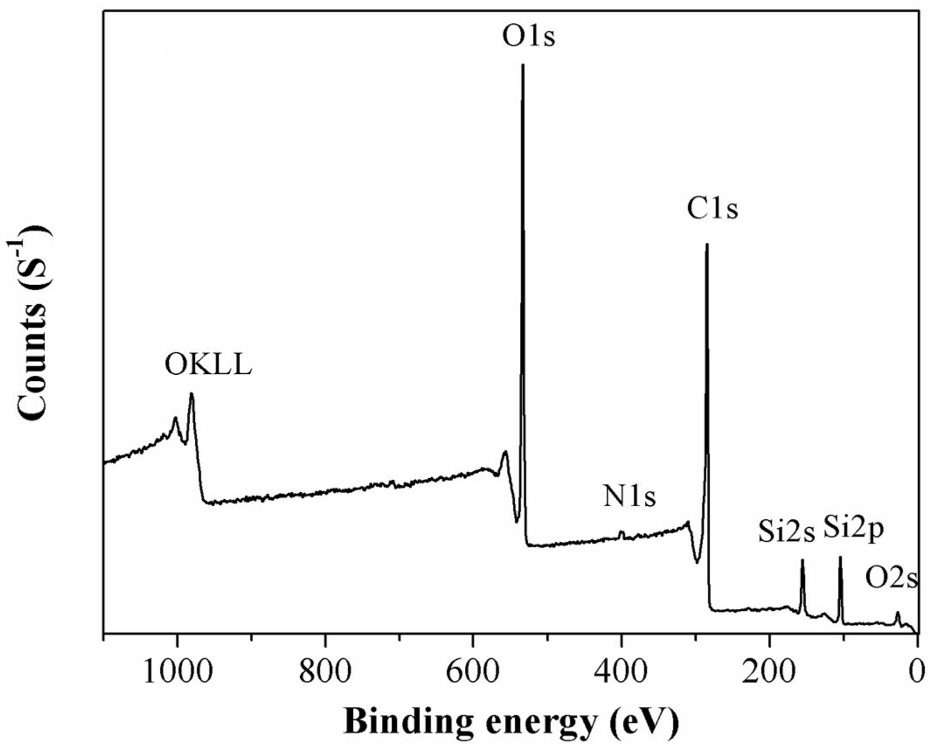

Fig. S1 Wide XPS spectrum of the MFAC.

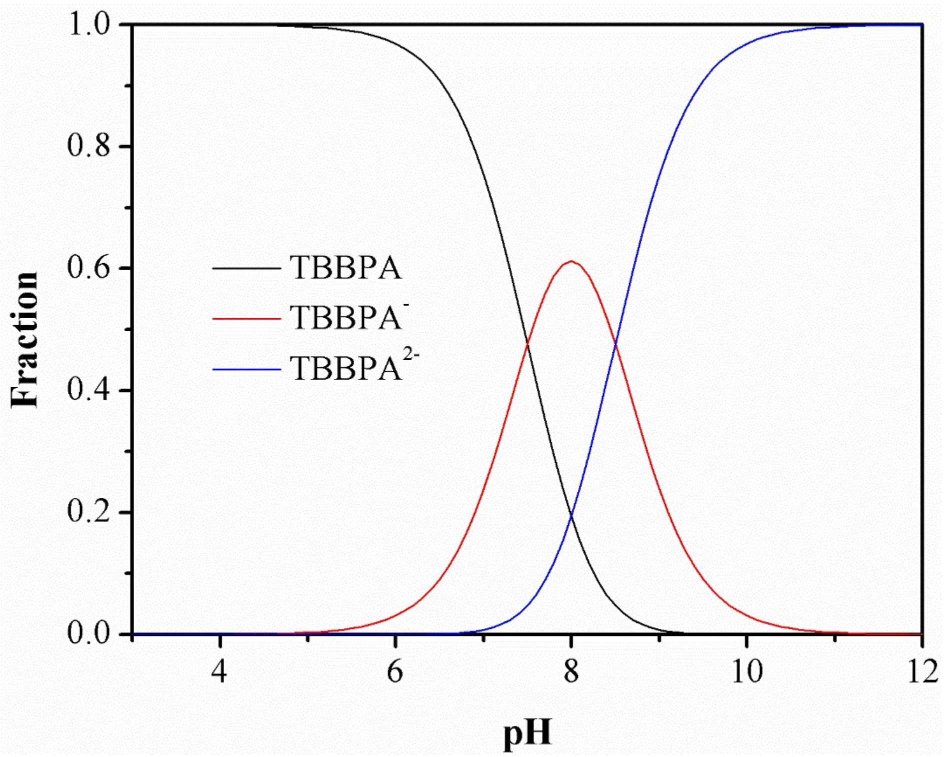

Fig. S2 Species distribution of TBBPA as a function of pH values.

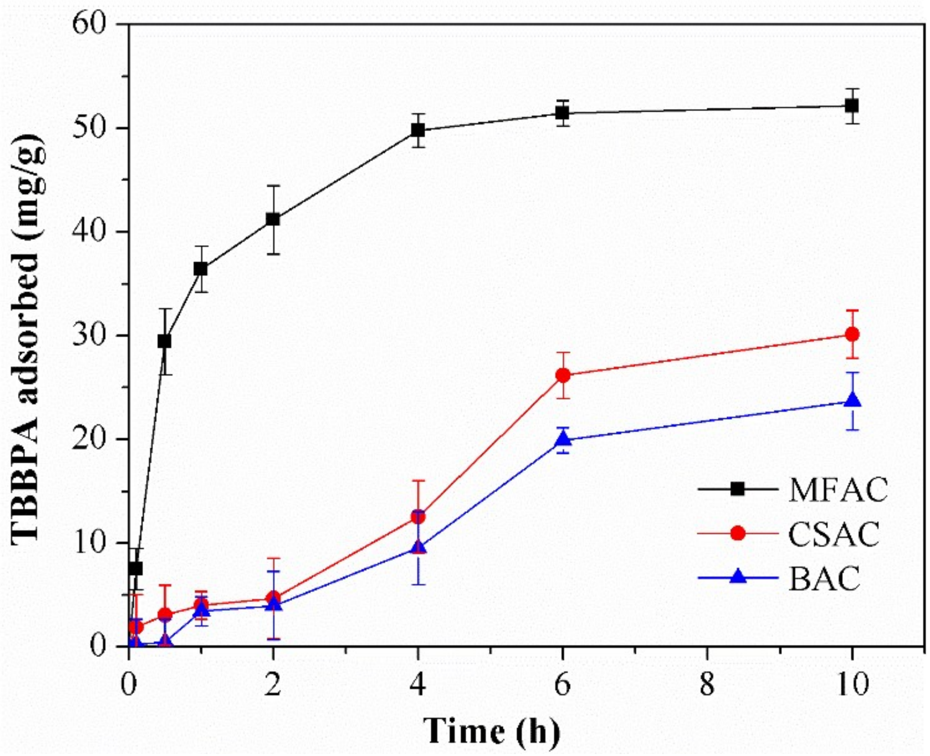

Fig. S3 Adsorption on TBBPA by different activated carbons (TBBPA = 10 mg/L, MFAC, commercial coconut shell activated carbon and bamboo activated carbon = 150.0 mg/L, T = 30 °C, pH = 9.0, t =10 h).
